# Alanine Racemase Mutants of *Burkholderia pseudomallei* and *Burkholderia mallei* and Use of Alanine Racemase as a Non-Antibiotic-Based Selectable Marker

**DOI:** 10.1371/journal.pone.0021523

**Published:** 2011-06-24

**Authors:** Sheryl L. W. Zajdowicz, Jessica Jones-Carson, Andres Vazquez-Torres, Michael G. Jobling, Ronald E. Gill, Randall K. Holmes

**Affiliations:** 1 Department of Microbiology, University of Colorado School of Medicine, Aurora, Colorado, United States of America; 2 Department of Biology, Metropolitan State College of Denver, Denver, Colorado, United States of America; East Carolina University School of Medicine, United States of America

## Abstract

*Burkholderia pseudomallei* and *Burkholderia mallei* are category B select agents and must be studied under BSL3 containment in the United States. They are typically resistant to multiple antibiotics, and the antibiotics used to treat *B. pseudomallei* or *B. mallei* infections may not be used as selective agents with the corresponding *Burkholderia* species. Here, we investigated alanine racemase deficient mutants of *B. pseudomallei* and *B. mallei* for development of non-antibiotic-based genetic selection methods and for attenuation of virulence. The genome of *B. pseudomallei* K96243 has two annotated alanine racemase genes (*bpsl2179* and *bpss0711*), and *B. mallei* ATCC 23344 has one (*bma1575*). Each of these genes encodes a functional enzyme that can complement the alanine racemase deficiency of *Escherichia coli* strain ALA1. Herein, we show that *B. pseudomallei* with in-frame deletions in both *bpsl2179* and *bpss0711*, or *B. mallei* with an in-frame deletion in *bma1575*, requires exogenous d-alanine for growth. Introduction of *bpsl2179* on a multicopy plasmid into alanine racemase deficient variants of either *Burkholderia* species eliminated the requirement for d-alanine. During log phase growth without d-alanine, the viable counts of alanine racemase deficient mutants of *B. pseudomallei and B. mallei* decreased within 2 hours by about 1000-fold and 10-fold, respectively, and no viable bacteria were present at 24 hours. We constructed several genetic tools with *bpsl2179* as a selectable genetic marker, and we used them without any antibiotic selection to construct an in-frame Δ*flgK* mutant in the alanine racemase deficient variant of *B. pseudomallei* K96243. In murine peritoneal macrophages, wild type *B. mallei* ATCC 23344 was killed much more rapidly than wild type *B. pseudomallei* K96243. In addition, the alanine racemase deficient mutant of *B. pseudomallei* K96243 exhibited attenuation versus its isogenic parental strain with respect to growth and survival in murine peritoneal macrophages.

## Introduction

*Burkholderia pseudomallei* and *Burkholderia mallei* are Gram-negative bacteria that cause meliodosis in humans and glanders in horses, respectively [1]. *B. mallei* is an equine pathogen [2], [3] that occasionally causes infections in humans. In contrast, *B. pseudomallei* is frequently isolated from the environment in tropical and subtropical areas [4], [5], and it is transmitted to humans by inhalation, ingestion, or direct contact [5]. The mortality rate for acute cases of human melioidosis exceeds 40%, and a percentage of the survivors may experience a relapse at a later time despite previous antibiotic treatment and apparent cure [5], [6].

Due to their ability to cause fatal infections, their intrinsic antibiotic resistance, and the lack of an approved vaccine, *B. pseudomallei* and *B. mallei* are considered to be potential biological warfare agents and are classified as Category B select agents in theUnited States (http://www.selectagents.gov/exclusions.html#hhsAgents). Under current select agent guidelines, antibiotic resistance markers cannot be used for experimental studies in *B. pseudomallei* or *B. mallei* if they confer resistance to antibiotics used to treat infections caused by these bacteria in humans or animals. To date, only antibiotic resistance genes that confer resistance to gentamicin, kanamycin, or zeocin are approved for use in *B. pseudomallei*, and only genes that encode resistance to kanamycin or zeocin are approved for use in *B. mallei*. Few genetic tools have been developed for use in *Burkholderia* spp., and they typically employ the antibiotic resistance genes described above as selectable markers [7], [8], [9], [10], [11], [12]. Because of these regulatory constraints, it is desirable to investigate the use of non-antibiotic-based selectable markers for the development of additional genetic tools for use in *Burkholderia* spp. Genes that encode alanine racemase, a key enzyme for bacterial cell wall biosythesis, have been used for this purpose in several other bacterial species.

Alanine racemases are pyridoxal 5′ phosphate-containing, homodimeric proteins that catalyze interconversion of l-alanine and d-alanine. d-alanine is an essential building block for biosynthesis of peptidoglycan in bacterial cell walls, and it is also found in lipotechoic acids of some Gram-positive bacteria [13], [14]. Alanine racemases have been well-studied in several bacteria, including *Escherichia coli* [15], *Listeria monocytogenes* [16], *Mycobacterium smegmatis* [17], *Pseudomonas putida* [18], *Salmonella enterica* serovar Typhimurium [19], *Corynebacterium glutamicum* [20], [21], *Lactobacillus plantarum* [22], and *Bacillus* spp. [23], [24], [25], [26]. Bacterial genomes usually contain either one or two alanine racemase genes. In bacteria with two alanine racemase genes, one is typically expressed constitutively and used for d-alanine biosynthesis, whereas the other is typically inducible and used for catabolism of d-alanine [27], [28], [29], [30]. In *E. coli*, *alr* encodes an alanine racemase that is constitutively expressed and has apparent anabolic activity, while *dadX* encodes an alanine racemase that is involved in l-alanine catabolism [15], [31]. A double knockout of both *dadX* and *alr* is required to produce an *E. coli* auxotroph that requires exogenous d-alanine for growth [31]. Bacteria that cannot produce alanine racemase exhibit a conditional lethal phenotype in the absence of exogenous d-alanine. An alanine racemase gene can therefore function as a selectable genetic marker in an alanine racemase deficient bacterial host growing in medium without exogenous d-alanine. Genes that encode alanine racemase have been used as an alternative to antibiotic resistance genes as selectable genetic markers in alanine racemase deficient variants of several bacterial species [20], [32], [33], [34]. Additionally, an alanine racemase deficient mutant of *L. monocytogenes* that exhibits defective growth in phagocytic cells and reduced virulence in animals has been investigated for potential use as a live attenuated vaccine against *L. monocytogenes* [16].

The characteristics of alanine racemase deficient mutants of *Burkholderia* spp. have not previously been evaluated. In this study, we identified the genes that encode alanine racemase in *B. pseudomallei* and *B. mallei*, constructed alanine racemase deficient mutant strains of both species, and compared the parental and alanine racemase deficient mutants of both species for growth and viability in the presence and absence of exogenous d-alanine, both *in vitro* and in murine peritoneal macrophages. Furthermore, we developed genetic tools with selectable alanine racemase markers to perform allelic exchange and gene complementation experiments in alanine racemase deficient host strains of *B. pseudomallei* and *B. mallei*. Finally, as proof of principle, we used these tools to construct an in-frame deletion in the *flgK* gene of *B. pseudomallei* and to complement the *flgK* mutant with a wild type *flgK* ^+^ allele on a replicating plasmid, thereby avoiding the need for antibiotics as selective agents.

## Results

### Identification of genes in *B. pseudomallei* and *B. mallei* that encode alanine racemases

**Figure 1:**
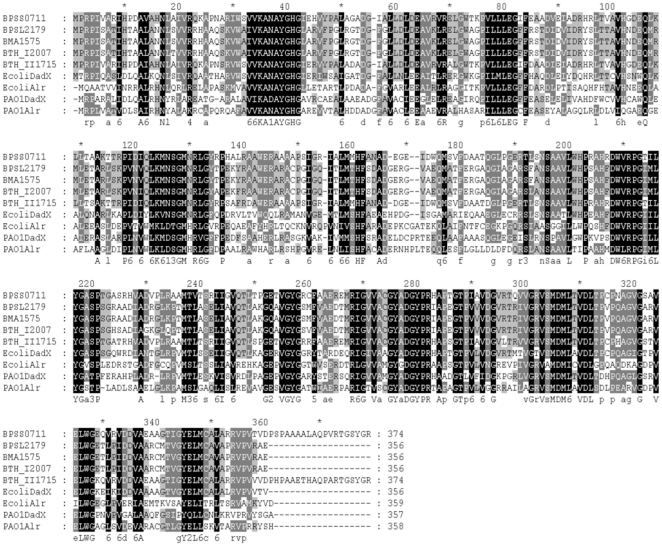
Alignment of amino acid sequences of alanine racemases from *B. pseudomallei* (BPSL2179 and BPSS0711), *B. mallei* (BMA1575), *B. thailandensis* (BTH_I2007 and BTH_II1715), *E. coli* (EcoliDadX and EcoliAlr), and *P. aeruginosa* (PAO1DadX and PAO1Alr). Residues that are identical in all of these alanine racemase enzymes are shown with a black background. Residues that are identical in 7 or 8 of these enzymes are shown with a dark grey background, and residues that are identical in 5 or 6 of these enzymes are shown with a light grey background.

Analysis of the genome sequence of *B. pseudomallei* strain K96243 [35] revealed two putative alanine racemase genes: *bpsl2179* on chromosome 1 which encodes a 356 amino acid protein, and *bpss0711* on chromosome 2 which encodes a 374 amino acid protein. Analysis of the genome sequence of *B. mallei* ATCC 23344 [36] revealed a single putative alanine racemase gene, designated *bma1575*. The coding sequences for *bma1575* and *bpsl2179* differ by only 3 nucleotides, and they encode identical proteins. We did not find a homologue of *bpss0711* in the genome sequence of *B. mallei* ATCC 23344. Figure 1 compares and aligns the predicted amino acid sequences for these putative alanine racemases from *B. pseudomallei* and *B. mallei* with the two reported alanine racemases per species from *Burkholderia thailandensis*, *E. coli* and *Pseudomonas aeruginosa* [37], [38], [39]. The identical proteins encoded by *bpsl2179* from *B. pseudomallei* and *bma1575* from *B. mallei* have 73% amino acid sequence similarity with the protein encoded by *bpss0711*from *B. pseudomallei* and 60–99% amino acid sequence similarity with the other alanine racemases shown in Figure 1.

We used genetic complementation tests in *E. coli* to demonstrate that *bpsl2179* and *bpss0711* from *B. pseudomallei* encode functional alanine racemases. Toward that end, we first constructed an alanine racemase deficient strain of *E. coli*, called ALA1, as described in Materials and Methods. *E. coli* ALA1 was unable to grow on LB agar or in LB broth unless d-alanine was added at ≥1 mM. In contrast, the parental strain of *E. coli* did not require added d-alanine for growth. When *E. coli* ALA1 was grown overnight at 37°C in LB broth with d-alanine and washed bacteria were sub-cultured in LB broth with or without d-alanine, the bacteria resumed normal growth in the LB broth with d-alanine, but they failed to grow and viability decreased progressively by about 1,000,000-fold over 8 hours in the LB broth without d-alanine (data not shown). Finally, introducing plasmid pET17*alr* into *E. coli* ALA1 restored the ability of *E. coli* ALA1 to grow in LB medium without added d-alanine. These findings demonstrate that *E. coli* ALA1 is auxotrophic for d-alanine and provide genetic evidence that it does not produce functional alanine racemase. Next, we constructed plasmids containing *bpsl2179* or *bpss0711* from *B. pseudomallei* (designated pCR2.1-TOPO® -*bpsl2179* and pCR2.1-TOPO®-*bpss0711*, respectively) and transformed them individually into *E. coli* ALA1. At 37°C on LB agar without d-alanine, visible growth of *E. coli* ALA1(pCR2.1-TOPO® -*bpsl2179*) transformants was detected after 15 hours, and visible growth of *E. coli* ALA1(pCR2.1-TOPO®-*bpss0711*) transformants was detected after 24 hours. We constructed a second *bpss0711* clone with a longer upstream sequence (Tables 2 and 3), designated pCR2.1-TOPO®-*bpss0711*-F2, and demonstrated that the growth of *E. coli* ALA1(pCR2.1-TOPO®-*bpss0711*-F2) transformants on LB agar without D-alanine was comparable to that of *E. coli* ALA1(pCR2.1-TOPO® -*bpsl2179*) transformants. We did not investigate further the molecular basis for the different properties of the pCR2.1-TOPO®-*bpss0711* and pCR2.1-TOPO®-*bpss0711*-F2 clones. We also showed that a pCR2.1-TOPO®-*bma1575* clone from *B. mallei* was able to complement the alanine racemase deficiency of *E. coli* ALA1. In contrast, the *E. coli* ALA1(pCR2.1-TOPO®) vector control did not grow under these conditions. Taken together, these genetic data demonstrate that *bpsl2179*, *bpss0711*, and *bma1575* encode functional alanine racemase proteins.

### Construction and characterization of *B. pseudomallei* and *B. mallei* alanine racemase deficient mutants

**Figure 2:**
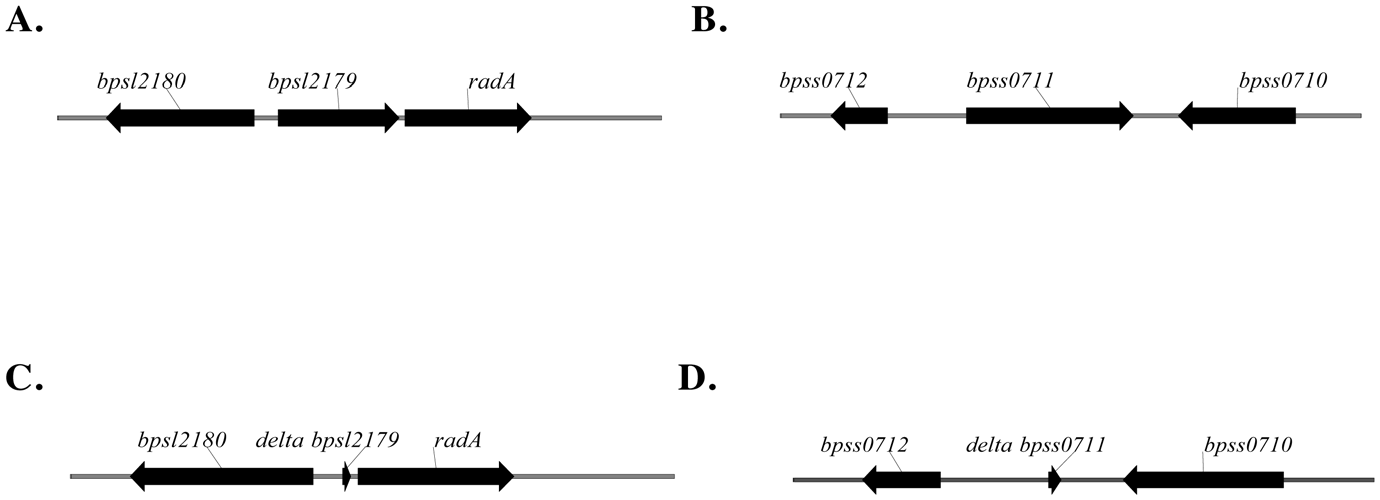
Organization of the *bpsl2179* and *bpss0711* loci in the large and small chromosome, respectively, of *Burkholderia pseudomallei* K96243. Panel A and Panel B show the relationship of the wild type *bpsl2179* and *bpss0711* alleles to their contiguous upstream and downstream genes. Panel C and Panel D show the locations and relative sizes of the in-frame deletion variants Δ*bpsl2179* and and Δ*bpss0711*, respectively, constructed in this study. The alanine racemase gene (*bma1575*) of *B. mallei* is contained within a region similar to that of the *bpsl2179* allele of *B. pseudomallei* shown in Panel A.

The arrangements of the *bpsl2179* locus in the large chromosome and the *bpss0711* locus in the small chromosome of *B. pseudomallei* K96243 are shown schematically in Fig. 2A and Fig. 2B, respectively. Both *bpsl2179* and *bpss0711* are predicted to be transcribed as single genes. The chromosomal loci carrying the corresponding Δ*bpsl2179* and Δ*bpss0711* in-frame deletion alleles described below are shown schematically in Fig. 2C and Fig. 2D, respectively.

**Figure 3:**
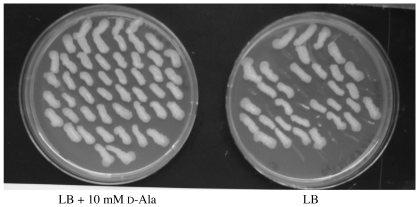
Screening of resolved co-integrants during construction of a Δ*bpsl2179/*Δ*bpss0711* double mutant of *B. pseudomallei*. Individual colonies of resolved co-integrants were picked from YT agar plates containing 15% sucrose and 10 mM d-alanine and inoculated in patches at comparable locations on LB agar plates with and without 10 mM d-alanine. Resolvants with the Δ*bpsl2179/*Δ*bpss0711* genotype grew only on the LB agar with d-alanine, and resolvants with the Δ*bpsl2179* genotype grew on LB agar with or without d-alanine.

**Figure 4:**
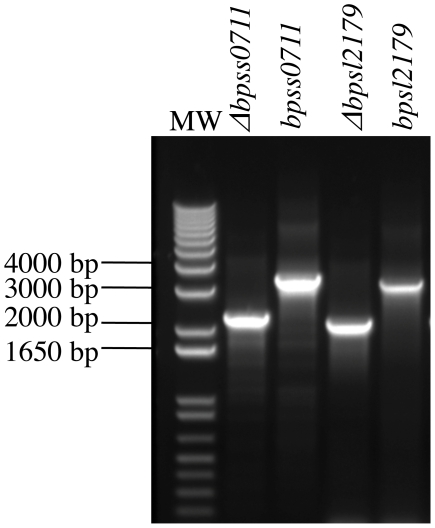
PCR confirmation of deletions in Δ*bpsl2179* and Δ*bpss0711* mutants of *B. pseudomallei*. Primers BPSL2179-up and BPSL2179-down were used to generate a 3108 bp fragment from strains carrying the wild type *bpsl2179* allele or a 2100 bp fragment from stains carrying the Δ*bpsl2179* allele. Primers BPSS0711-up and BPSS0711-down were used to generate a 3247 bp fragment from strains carrying the wild type *bpss0711* allele or a 2188 bp fragment from strains carrying the Δ*bpss0711* allele. Lane labels and samples analyzed are as follows: MW, DNA ladder; Δ*bpss0711*, amplicon from strain carrying the Δ*bpsl2179* mutant allele; *bpss0711*, amplicon from strain carrying the wild type *bpss0711 allele*; Δ*bpsl2179*, amplicon from strain carrying the Δ*bpsl2179* mutant allele; and *bpsl2179*, amplicon from strain carrying the wild type *bpsl2179* allele.

We used *in vitro* methods to construct mutant alleles, designated Δ*bpsl2179* and Δ*bpss0711*, with in-frame deletions corresponding to 99% of the coding regions of *bpsl2179* and *bpss0711*, respectively. These mutant alleles were substituted, either singly or together, for the corresponding wild type alleles in the chromosomes of *B. pseudomallei* strain K96243 and *B. pseudomallei* strain 1026b by using the pMo130 allelic exchange system previously described [12]. In both of these *B. pseudomallei* strains, the resulting single mutants carrying either Δ*bpsl2179* or Δ*bpss0711* grew normally on LB agar without added d-alanine, but the Δ*bpsl2179/*Δ*bpss0711* double mutant required exogenous d-alanine for growth. Fig. 3 illustrates the patch tests that we used to distinguish individual resolved co-integrants with the Δ*bpsl2179/*Δ*bpss0711* double mutant genotype that grow only on LB agar containing d-alanine from individual resolved co-integrants with the Δ*bpsl2179* single mutant genotype that do not require d-alanine for growth. Our results showed that a single wild type allele of either *bpsl2179* or *bpss0711* is sufficient for normal growth of *B. pseudomallei* on LB agar without added d-alanine, and they showed that no gene except *bpsl2179* or *bpss0711* directs production of functional alanine racemase in *B. pseudomallei* strain K96243 or strain 1026b. In contrast, a single in-frame deletion at the *bma1575* locus of *B. mallei* ATCC 23344, introduced by allelic exchange using the highly homologous pMo130Δ *bpsl2179* clone, conferred a stringent growth requirement for exogenous d-alanine. This finding indicates that *bma1575* is the only gene in *B. mallei* ATCC 23344 that directs production of alanine racemase. The presence of the appropriate wild type or in-frame deletion variant of *bpsl2179* or *bpss0711* in each newly constructed mutant was confirmed by PCR using primers located upstream and downstream of the appropriate gene, as illustrated in Fig. 4. For all subsequent experiments, the term “alanine racemase deficient mutants" refers to a Δ*bpsl2179/*Δ*bpss0711* double mutant for *B. pseudomallei* strain K96243 or *B. pseudomallei* strain 1026b, and to a Δ*bma1575* single mutant for *B. mallei* strain ATCC 23344.

To demonstrate complementation of the functional defect in the alanine racemase deficient mutants of *B. pseudomallei* and *B. mallei*, the wild type *bpsl2179* gene was first cloned into the replicative plasmid pMo168 [12] in place of the *aphA* cassette, yielding pALR-comp. The pALR-comp plasmid was then introduced by conjugation into the alanine racemase deficient mutant strains of *B. pseudomallei* K96243, *B. pseudomallei* 1026b, and *B. mallei* ATCC 23344. Transconjugant colonies were recovered on LB agar containing zeocin or polymyxin B. The presence of pALR-comp in transconjugant colonies was confirmed by detecting the XylE reporter enzyme encoded by pALR-comp, and complementation of the alanine racemase deficiency was confirmed by the ability of the transconjugants to grow with or without the addition of d-alanine.

**Figure 5:**
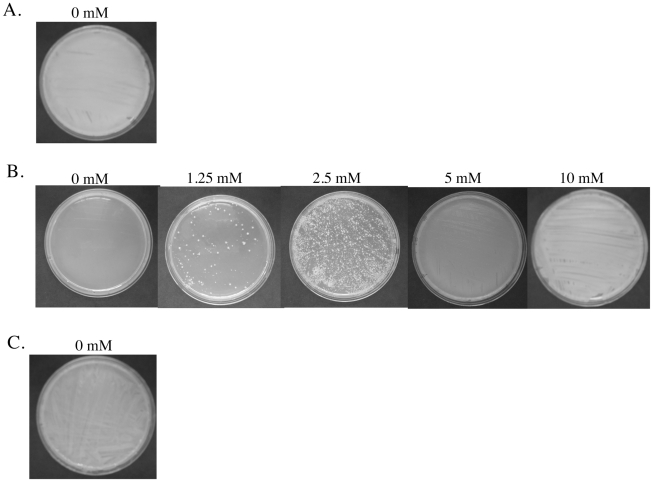
Determination of d-alanine concentration needed to support growth of the Δ*bpsl2179/*Δ*bpss0711* alanine racemase deficient mutant of *B. pseudomallei* K96243 on LB agar medium. Overnight cultures of the wild type, Δ*bpsl2179/*Δ*bpss0711* mutant, and Δ*bpsl2179/*Δ*bpss0711* mutant complemented with pALR-comp were inoculated into LB broth containing 10 mM d-alanine. Cultures were grown to an OD_600 nm_ = 0.2 and plated onto LB agar without d-alanine (0 mM) or with d-alanine at concentrations from 1.25 mM to 10 mM as indicated. Panel A: *B. pseudomallei* K96243. Panel B: The Δ*bpsl2179/*Δ*bpss0711* mutant of *B. pseudomallei* K96243. Panel C.: The Δ*bpsl2179/*Δ*bpss0711* mutant of *B. pseudomallei* K96243 complemented with plasmid pAlr-comp. Comparable results were obtained with isogenic wild type, alanine racemase deficient, and complemented alanine racemase deficient strains of *B. pseudomallei* 1026b and *B. mallei* ATCC 23344 (data not shown).

To determine the minimal concentration of d-alanine necessary to support growth of the *Burkholderia* alanine racemase deficient mutants, samples from log phase cultures of each mutant, wild type, or complemented strain were spread onto LB agar with d-alanine at various concentrations from 0 to 10 mM, and the plates were inspected for bacterial growth during subsequent incubation at 37°C. Fig. 5 shows the results for the *B. pseudomallei* K96243-derived strains. After 24–36 hr, the wild type parental strain (Fig. 5A) and the complemented mutant (Fig. 5C) showed heavy confluent growth on medium without d-alanine. In contrast, the Δ*bpsl2179/*Δ*bpss0711* mutant showed no growth on medium without d-alanine, scattered colonies on medium with 1.25 mM d-alanine, sub-confluent growth on medium with 2.5 mM d-alanine, and confluent growth on medium with 5 or 10 mM d-alanine (Fig. 5B). Comparable results were observed with the parental, mutant and complemented strains derived from *B. pseudomallei* 1026b and *B. mallei* ATCC 23344 (data not shown). It is noteworthy that the concentration of d-alanine needed to support growth of the alanine racemase deficient mutants of *B. pseudomallei* and *B. mallei* was significantly greater than the concentration of d-alanine required to support growth of *E. coli* ALA1.

**Figure 6:**
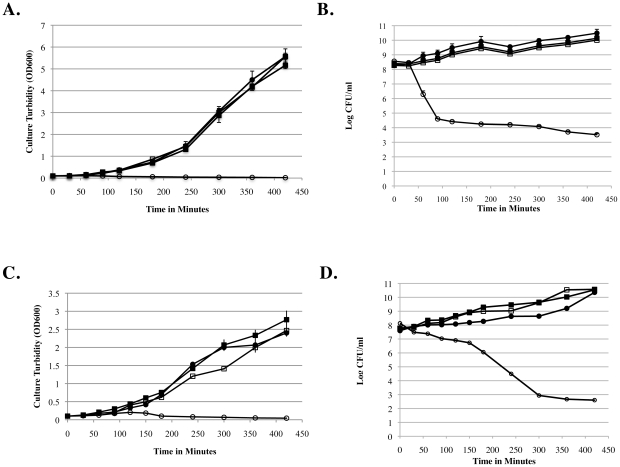
Alanine racemase deficient mutants of *B. pseudomallei* and *B. mallei* grew normally in LB broth with 10 mM d-alanine, but they exhibited growth arrest and rapidly lost viability in LB broth without d-alanine. In contrast, the isogenic parental strains of *B. pseudomallei* and *B. mallei* grew normally in LB broth either with or without added d-alanine. In each panel, a closed square (▪) represents the wild type strain grown in the presence of d-alanine; an open square (□) represents the wild type strain grown without d-alanine; a closed circle (•) represents the alanine racemase deficient mutant grown in the present of d-alanine; and an open circle (○) represents the alanine racemase deficient mutant grown without d-alanine. Panel A: Turbidity of cultures of wild type and mutant strains of *B. pseudomallei* K96243. Panel B: Viability of bacteria in cultures of wild type and mutant strains of *B. pseudomallei* K96243. Panel C: Turbidity of cultures of wild type and mutant strains of *B. mallei* ATCC 23344. Panel D: Viability of bacteria in cultures of wild type and mutant strains of *B. mallei* ATCC 23344. Results with wild type and mutant strains of *B. pseudomallei* 1026b (not shown) were similar to those obtained with wild type and mutant strains of *B. pseudomallei* K96243.

To assess the effects of d-alanine deprivation on growth and viability of the wild type and alanine racemase deficient variants of *B. pseudomallei* and *B. mallei* as a function of time, we collected bacteria from cultures grown overnight in LB broth containing 10 mM d-alanine, washed the bacteria with LB broth, and transferred inocula into LB broth subcultures with or without 10 mM d-alanine. We measured turbidity and viable counts in samples taken periodically from each subculture during the first seven hours (Fig. 6) and again at 24 hours. The alanine racemase deficient mutants of *B. pseudomallei* K96243 and *B. mallei* ATCC 23344 exhibited growth arrest in LB broth without d-alanine (Fig. 6A and Fig. 6C), but the growth of each mutant in LB broth with d-alanine was comparable to the growth of its wild type parental strain in LB broth with or without d-alanine. Under the conditions that permitted growth, the doubling time for the wild type and mutant *B. mallei* strains was about 48 minutes, and the doubling time for the wild type and mutant *B. pseudomallei* strains was about 36 minutes. Furthermore, each alanine racemase deficient mutant, but not its isogenic parental strain, lost viability progressively with increasing incubation time in the LB broth without d-alanine (Fig. 6B and Fig. 6D). During the first two hours without d-alanine, the alanine racemase deficient mutant of *B. pseudomallei* K96243 lost viability more rapidly than the alanine racemase deficient mutant of *B. mallei* strain ATCC 23344 (∼4 log_10_ decrease vs. ∼1 log_10_ decrease, respectively). After 7 hours without d-alanine, viability declined by about 5 log_10_ for both of the alanine racemase deficient mutants. After 24 hours without d-alanine, no viable bacteria were recovered from cultures of either of the alanine racemase deficient mutants (data not shown). Results with the wild type and alanine racemase deficient mutant of *B. pseudomallei* 1026b were comparable to those shown above for the wild type and alanine racemase deficient mutant of *B. pseudomallei* K96243, respectively (data not shown).

**Figure 7:**
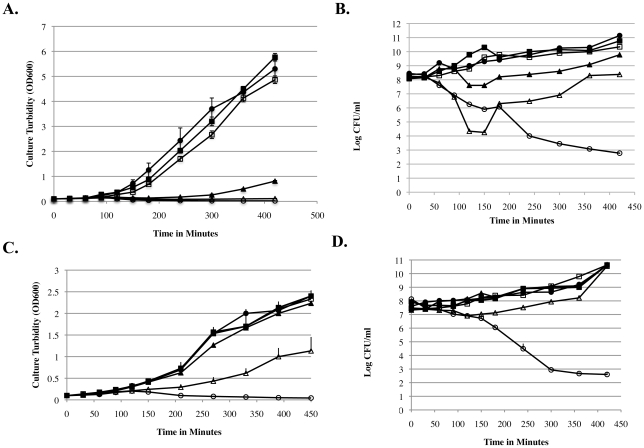
Ability of alanine racemase deficient mutants of *B. pseudomallei* or *B. mallei* to recover from varying periods of d-alanine deprivation. The alanine racemase deficient mutants of *B. pseudomallei* K96243 and *B. mallei* ATCC 23344 were grown overnight in LB broth containing 10 mM d-alanine. Inocula from each culture were transferred into sets of six replicate subcultures in LB broth. Supplemental d-alanine at a final concentration of 10 mM was added to separate LB broth subcultures of each mutant at 0, 30, 60, 90, or 120 minutes post-inoculation, respectively, and the final subculture received no added d-alanine. Culture turbidity (OD_600 nm_) and viability was measured for each subculture periodically up to 420 minutes. In each panel, an open circle (○) represents the subculture without added d-alanine; a closed circle (•) represents of the subculture with d-alanine added at time zero; a closed square (▪) represents the subculture with d-alanine after added at 30 min; an open square (□) represents the subculture with d-alanine added at 60 min; a closed triangle (▴) represents the subculture with d-alanine added at 90 min; and an open triangle (▵) represents the subculture with d-alanine added at 120 min. Panel A; Turbidity of subcultures of the alanine racemase deficient mutant of *B. pseudomallei* K96243. Panel B: Viable counts in subcultures of the alanine racemase deficient mutant of *B. pseudomallei* K96243. Panel C: Turbidity of subcultures of the alanine racemase deficient mutant of *B. mallei* ATCC 23344. Panel D: Viable counts in subcultures of the alanine racemase deficient mutant of *B. mallei* ATCC 23344. Results for the alanine racemase deficient mutant of *B. pseudomallei* K96243 are comparable to the results obtained with the alanine racemase deficient mutant of *B. pseudomallei* 1026b (data not shown).

To investigate how efficiently each of the alanine racemase deficient mutants of *B. pseudomallei* or *B. mallei* can recover from d-alanine deprivation for varying periods of time, we grew each mutant overnight in LB broth containing 10 mM d-alanine and then transferred washed bacteria into six identical subcultures containing LB broth alone. At time 0, 30, 60, 90, and 120 minutes post inoculation, 10 mM d-alanine was added to separate subcultures, and the sixth subculture served as a control without added d-alanine. Samples were withdrawn from each subculture periodically for a total of 7 hours, and each sample was examined for turbidity and viable counts (Fig. 7). For the alanine racemase deficient mutant of *B. pseudomallei* strain K96243, growth resumed after a few minutes when d-alanine was added back to the cultures at 30 or 60 minutes (Fig. 7A). In contrast, d-alanine deprivation for 90 minutes caused a lag period of at least 2 hours before growth resumed, and d-alanine deprivation for 120 minutes caused growth arrest to the end of the 7-hour observation period (Fig. 7A). d-alanine deprivation for 30 or 60 minutes did not cause a substantial decrease in viability of the alanine racemase deficient mutant of *B. pseudomallei* strain K96243 (Fig. 7B). In contrast, 90 minutes of d-alanine deprivation resulted in ∼1 log_10_ decrease in viability, and 120 minutes of d-alanine deprivation caused ∼3 log_10_ decrease in viability. Bacteria that were viable after each period of d-alanine deprivation resumed growth shortly after d-alanine was added back to the medium. Similar results were obtained with the alanine racemase deficient mutant of *B. pseudomallei* strain 1026b (data not shown). For the alanine racemase deficient mutant of *B. mallei*, prolonged growth retardation and a transient decrease in viability occurred with d-alanine deprivation for 120 minutes but not with d-alanine deprivation for 90 minutes or less (Figs. 7C and 7D).

### Intracellular survival of wild type and alanine racemase deficient strains of *B. pseudomallei* and *B. mallei* in periodate-elicited murine peritoneal macrophages

**Figure 8:**
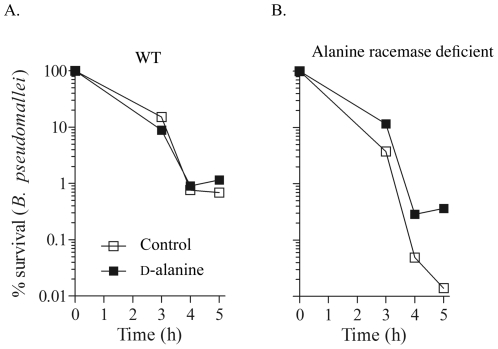
Survival of wild type and isogenic alanine racemase deficient mutant strains of *B. pseudomallei* within murine macrophages. A) Survival of wild type *B. pseudomallei* K96243 in murine macrophages in RPMI^+^ medium containing 250 µg/ml of kanamycin with (▪) or without (□) 5 mM d-alanine. B) Survival of the isogenic alanine racemase deficient mutant strain Δ*bpsl2179*/Δ*bpss0711* in murine macrophages in RPMI^+^ medium containing 250 µg/ml of kanamycin with (▪) or without (□) 5 mM d-alanine.

**Figure 9:**
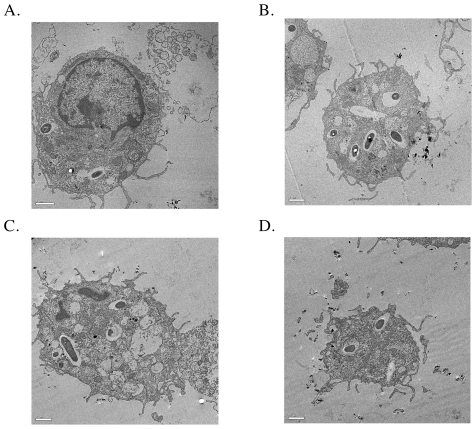
Phagocytosis of wild type and isogenic alanine racemase deficient mutant strains of *B. pseudomallei* and *B. mallei* by murine macrophages. Immediately after the phagocytosis phase of macrophage killing assays, the macrophages were fixed and examined by transmission electron microscopy for the presence of intracellular bacteria. A) Wild type *B. pseudomallei* K96243; B) Isogenic alanine racemase deficient *B. pseudomallei* K96243; C) Wild type *B. mallei* ATCC 23344; D) Isogenic alanine racemase deficient *B. mallei* ATCC 23344.

*B. pseudomallei* is able to survive in non-phagocytic cells and phagocytic cells [40] and *B. mallei* can survive in macrophages [41]. Due to the rapid decrease in viability of the alanine racemase deficient mutants under conditions of d-alanine deprivation and the requirement for high concentrations of D-alanine to support normal growth of these mutants, we tested whether the alanine racemase deficient mutant strains of *B. pseudomallei* and *B. mallei* would exhibit decreased survival in phagocytic cells in comparison with their isogenic parental strains. First, we infected murine peritoneal macrophages with wild type or alanine racemase deficient strains of *B. pseudomallei* K96243. Bacteria were added to the macrophage cultures in RPMI^+^ medium supplemented with 5 mM d-alanine, and the infected cultures were incubated for 3 hours to permit phagocytosis of *B. pseudomallei*. The cultures were then washed with media containing kanamycin plus or minus 5 mM d-alanine, and the viable intracellular bacteria were enumerated at times 0, 3, 4, and 5-hours in sets of 5 replicate cultures. The % survival was expressed as (cfu at t_n_/cfu at t_0_) ×100. For wild type *B. pseudomallei* K96243, survival of intracellular bacteria was approximately 1% after 5 hours, and survival was not affected by the presence or absence of 5 mM d-alanine in the culture medium (Fig. 8A). In contrast, intracellular survival of the alanine racemase deficient mutant of *B. pseudomallei* K96243 after 5 hours in medium without d-alanine was only 0.01%, compared to about 0.5% in medium containing 5 mM d-alanine (Fig. 8B). To confirm the internalization of the wild type and mutant bacteria, we collected macrophages after the initial 3-hour period of contact with bacteria and analyzed them by transmission electron microscopy (Fig. 9). Comparable numbers of intracellular bacteria were observed for the wild type and mutant bacterial strains (Fig. 9, Panels A and B). Taken together, these findings demonstrate a substantially decreased ability of the alanine racemase deficient mutant of *B. pseudomallei* K96243 to survive within murine peritoneal macrophages unless a high concentration of d-alanine was present during the killing assay.

**Figure 10:**
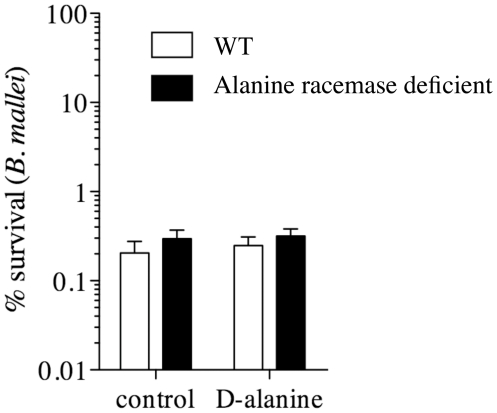
Survival of wild type and isogenic alanine racemase deficient mutant strains of *B. mallei* within murine macrophages. Murine peritoneal periodate-elicited macrophages were infected in vitro with wild type or alanine racemase deficient mutant strains of *B. mallei*. After 2 h of phagocytosis in d-alanine-supplemented RPMI^+^ medium, the macrophages were washed with prewarmed RPMI^+^ medium containing 6 µg/ml gentamicin with or without 5 mM d-alanine. The surviving intracellular bacteria were enumerated after 4 h of culture. The % survival was enumerated by (cfu t_n_/cfu t_0_)100.

Next, we compared the wild type and alanine racemase deficient strains of *B. mallei* in macrophage killing assays. Murine peritoneal macrophages were infected with the wild type or alanine racemase deficient mutant strain of *B. mallei* ATCC23344 under conditions similar to those described above for *B. pseudomallei*. After 2 hours of phagocytosis in d-alanine-supplemented RPMI^+^ medium, the macrophages were washed with pre-warmed RPMI^+^ medium containing 6 µg/ml gentamicin with or without 5 mM D-alanine. Electron microscopy demonstrated comparable numbers of wild type and mutant bacteria within macrophages immediately after the 2-hour phagocytosis phase of the killing assays (Fig. 9, Panels C and D). Survival of the intracellular bacteria was determined after an additional 4 hours of incubation. In contrast to the previous results for *B. pseudomallei*, both wild type *B. mallei* and the isogenic alanine racemase deficient mutant were equally susceptible to killing by macrophages, and enhanced killing of the alanine racemase deficient mutant was not observed in medium without d-alanine (Fig. 10). In conclusion, the alanine racemase deficient mutant of *B. mallei* did not exhibit an increased susceptibility to intracellular killing in macrophages under these experimental conditions.

### The use of alanine racemase as a selectable genetic marker

To demonstrate the use of alanine racemase as selectable genetic marker for construction of mutant strains of *Burkholderia spp.*, we first replaced the *aphA* gene in the allelic exchange vector pMo130 [12] with *bpsl2179* from *B. pseudomallei* K96243 to generate pAlr-allex. Then, as proof of principle, we used the pAlr-allex vector system to construct a Δ*flgK* mutant without use of antibiotics for selection. This experiment was based on results of a previous study demonstrating that deletion of *flgK*, which encodes the flagellar protein FlgK, causes loss of motility in *B. pseudomallei* [12]. By cloning Δ*flgK* from pMo146 [12] into pAlr-allex, we targeted the *flgK* gene of *B. pseudomallei* for deletion. The resulting plasmid designated pAlr-allex-Δ*flgK* was introduced by conjugation into the alanine racemase deficient *B. pseudomallei* strains K96243 Δ*bpss0711/*Δ*bpsl2179* and 1026b Δ*bpss0711/*Δ*bpsl2179*. Transconjugants were selected by growth on LB agar medium without added D-alanine and confirmed by detection of XylE activity. Following counter-selection on sucrose, some of the resolved transconjugants of *B. pseudomallei* strains K96243 and 1026b exhibited both a loss of motility and a growth requirement for d-alanine, consistent with their Δ*flgK*Δ*bpss0711*Δ*bpsl2179* genotypes (data not shown).

To demonstrate use of alanine racemase as a selectable genetic marker for complementation tests, we cloned the wild type *flgK^+^* allele into pAlr-comp and introduced the resulting pAlr-comp-*flgK^+^* plasmid by conjugation into the Δ*flgK/*Δ*bpss0711/*Δ*bpsl2179* mutants of *B. pseudomallei* strains K96243 and 1026b described above. Transconjugants were selected by growth on LB agar medium lacking d-alanine and confirmed by detection of XylE activity. Introduction of pAlr-comp-*flgK^+^* into these non-motile mutants of *B. pseudomallei* restored motility to wild type levels in addition to restoring the ability of the mutants to grow without added d-alanine (data not shown). These experiments using pAlr-allex and pAlr-comp established proof of principle that alanine racemase can be used as a selectable marker for construction of in-frame mutants by allelic exchange and for *in trans* complementation in pathogenic *Burkholderia* spp.

## Discussion

In the United States, the study of *B. pseudomallei* and *B. mallei* is highly regulated because they are classified as category B select agents. Furthermore, few antibiotic resistance determinants are approved as selectable markers for use in genetic studies of *B. pseudomallei* and *B. mallei* [5], [42]. Previous studies have identified several non-antibiotic selectable markers for use in genetic studies of bacteria. Most are based on the lethal consequences of inactivating an essential gene, and preserving viability by providing a wild type allele of the inactivated essential gene to complement the lethal phenotype. One such system used a *thyA* mutant of *Lactococcus lactis* that cannot survive without an exogenous source of thymidine or thymine unless *thyA* ^+^ is introduced on a replicating plasmid or by another genetic method [43], [44]. In *Salmonella enterica* serovar T*yphimurium* and *P. aeruginosa*, a comparable system was based on inactivation of the *asd* gene encoding aspartate β-semialdehyde dehydrogenase, an enzyme required for diaminopimelic acid production and peptidoglycan synthesis in these bacterial species [45], [46]. Without diaminopimelic acid, *asd* mutants will lyse, whereas *asd* mutants complemented with an *asd* ^+^ allele will survive and grow normally. In *C. glutamicum* and *L. monocytogenes* [20], [33], [34], inactivation of the gene encoding alanine racemase, an enzyme required for production of D-alanine and for peptidoglycan biosynthesis, was shown to be lethal unless d-alanine was added to the growth medium or the genetic defect was complemented by the presence of a wild type allele of the alanine racemase gene.

In the present study, we constructed and characterized alanine racemase mutants of *B. pseudomallei* and *B. mallei* and demonstrated that they exhibit a stringent requirement for exogenous d-alanine for growth and viability. In *B. pseudomallei*, it was necessary to inactivate both *bpsl2179* and *bpss0711* to produce an alanine racemase deficient phenotype, whereas in *B. mallei* inactivation of *bma1575* alone was sufficient to produce an alanine racemace deficient phenotype. Other bacterial species whose genomes encode two different but functional alanine racemases include *S. enterica* serovar Typhimurium, *P. aeruginosa*, and *E. coli* [19], [27], [47], [48], [49]. In contrast, *C. glutamicum* [20] and *Lactobacillus plantarum* [22] are representative of bacterial species whose genomes encode only one functional alanine racemase.

In this study, we showed that a gene encoding alanine racemase can be used effectively as a selectable marker for genetic manipulations in alanine racemase deficient parental strains of pathogenic *Burkholderia* spp. We constructed an allelic exchange vector with a selectable alanine racemase gene and used it to generate a *ΔflgK* mutation in two different alanine racemase deficient reference strains of *B. pseudomallei*. In addition, we constructed a replicative plasmid carrying wild type alleles of the alanine racemase gene *bpsl2179* and the *flgK* gene and showed that it could complement the *ΔflgK* mutation in an alanine racemase deficient strain and restore its ability to grow in the absence of exogenous d-alanine. The pAlr-allex and pAlr-comp plasmids described in this study are significant additions to the current repertoire of tools for use in genetic studies of pathogenic *Burkholderia* spp.

We also demonstrated that an alanine racemase deficient mutant of *B. pseudomallei* K96243 lost viability at a much faster rate than its isogenic parental strain in murine peritoneal periodate-elicited macrophages growing in cell culture medium without added d-alanine. However, the addition of 5 mM d-alanine to the cell culture medium allowed the alanine racemase deficient mutant to survive intracellularly as well as its wild type parental strain. In another published study, an alanine racemase deficient (*dal dat*) mutant of *L. monocytogenes* also exhibited growth attenuation in macrophages and required exogenous d-alanine in the culture medium to achieve levels of intracellular survival comparable to the wild type parental strain [16]. *L. monocytogenes* lacking alanine racemase failed to elicit an immune response in mice when it was inoculated without added d-alanine. In contrast, when it was inoculated together with d-alanine, the mice developed a protective immune response and survived a subsequent challenge with a dose of wild type *L. monocytogenes* that was lethal for un-immunized mice [16]. The authors proposed that the addition of d-alanine enabled the attenuated strain to undergo limited replication in the inoculated mice that was sufficient to elicit a protective immune response [16]. They also showed that few viable bacteria remained in the mouse tissues after 1–2 days [16], presumably because depletion of the inoculated d-alanine by d-amino acid oxidases of the mouse [50], [51], [52], [53], [54] resulted in rapid death of the inoculated bacteria and their progeny. Our alanine racemase deficient mutant of *B. mallei* did not exhibit attenuation under the conditions used for our experiments. Because wild type *B. mallei* grows more slowly than *B. pseudomallei* and is killed more rapidly than *B. pseudomallei* in macrophages, it seems likely that the *B. mallei* mutant did not grow enough within the macrophages to permit d-alanine deprivation to contribute significantly to the overall loss of viability. Since there is currently no vaccine for *B. pseudomallei* or *B. mallei*, the use of an attenuated strain as a potential vaccine is appealing. Additional studies will be needed to examine the degree of attenuation of our alanine racemase deficient mutants of *B. pseudomallei* and *B. mallei* in normal and immunocompromised animals and to test their ability to stimulate protective immune responses after they are inoculated with or without added d-alanine, as described above for alanine racemase deficient mutants of *L. monocytogenes*. Such experiments are beyond the scope of this study and will require animal facilities approved for use with select agents that are not available at our institution. Recently, Propst et al. [55] reported that a *ΔpurM* mutant of *B. pseudomallei* is attenuated both in immunocompetent and immunodeficient animals and could be considered for possible exclusion from select agent classification. Additional studies will be needed to demonstrate whether alanine racemase deficient mutants of *B. pseudomallei* and *B. mallei* may also be appropriate candidates for possible exclusion from select agent classification. Strongly attenuated mutants of *Bacillus anthracis*, *Brucella abortus*, *Coxiella burnetii*, *Francisella tularensis*, and *Yersinia pestis* have already been excluded from select agent classification (http://www.selectagents.gov/exclusions.html#hhsAgents) [55].

There are many ways that purine- or D-alanine requiring mutants such as those described above could be used to facilitate future genetic studies with *B. pseudomallei* or *B. mallei*. Possible exclusion from select agent classification would enable such strains to be used for genetic manipulations under BSL2 containment rather than BSL3 containment. This would greatly simplify the technical procedures required for construction of mutants with targeted inactivating mutations in specific non-essential genes, or construction of libraries of mutants with random inactivating mutations in any non-essential genes. Mutants of interest could then be placed under BSL3 conditions, and the effects on virulence of inactivating mutations in individual genes could be tested after the purine or D-alanine requirement of the parental strain was eliminated by introduction of an appropriate complementing gene.

## Materials and Methods

### Bacterial strains, growth conditions, and media

All bacterial strains and plasmids used in this study are listed in Table 1 and Table 2, respectively, and all primers used for cloning in this study are listed in Table 3. All primers were purchased from Integrated DNA Technologies (Coralville, IA). All *E. coli* strains were grown in Luria-Bertani (LB) media at 30°C or 37°C as described in the text. All experiments involving live cultures of *B. pseudomallei* or *B. mallei* were performed in the BSL3 facility at the University of Colorado School of Medicine. All *Burkholderia* strains were grown in LB broth at 37°C unless otherwise stated. Kanamycin, when appropriate for plasmid selection, was added at a concentration of 50 µg/ml unless otherwise noted. d-alanine, when needed, was added to LB media at a concentration of 1 mM for experiments with *E. coli* and at 10 mM for experiments *B. pseudomallei* or *B. mallei*. For resolution of co-integrants by sucrose counter-selection, *Burkholderia* co-integrants were grown in YT broth, which was made by dissolving 10 g yeast extract and 10 g tryptone in 1 l of H_2_O containing 15% sucrose as described by Hamad *et al* [12].

**Table 1:** Bacterial strains used in this study.

Strains	Relevant features and use	Reference
*B. pseudomallei* K96243	Human Clinical isolate; Km^R^Gm^R^Zeo^R^ Pb^R^	[35]
*B. pseudomallei* 1026b	Human Clinical isolate; Km^R^ Gm^R^ Zeo^R^ Pb^R^	[10]
*B. mallei* ATCC 23344	Human Clinical isolate; Km^S^ Gm^S^ Zeo^S^ Pb^R^	[62]
*B. pseudomallei* K96243 Δ*bpss0711*	Unmarked deletion of *bpss0711*	This study
*B. pseudomallei* K96243 Δ*bpsl2179*	Unmarked deletion of *bpsl2179*	This study
*B. pseudomallei* K96243 Δ*bpss0711*Δ*bpsl2179*	Unmarked deletions of *bpss0711 and bpsl2179*, d-alanine requiring	This study
*B. pseudomallei* K96243 Δ*bpss0711*Δ*bpsl2179*+pAlr-comp	Unmarked deletions of *bpss0711 and bpsl2179* carrying complementation replicating vector pAlr-comp; no d-alanine requirement	This study
*B. pseudomallei* K96243 Δ*bpss0711*Δ*bpsl2179*Δ*flgK*	Unmarked deletions of *bpss0711*, *bpsl2179*, and *flgK*, d-alanine requiring and non-motile	This study
*B. pseudomallei* K96243 Δ*bpss0711*Δ*bpsl2179*Δ*flgK*+pAlr-comp*flgK*	Unmarked deletions of *bpss0711*, *bpsl2179*, and *flgK* carrying complementation replicating vector pAlr-comp *flgK*; no d-alanine requirement and motile	This study
*B. pseudomallei* 1026b Δ*bpss0711*Δ*bpsl2179*	Unmarked deletions of *bpss0711 and bpsl2179*, d-alanine requiring	This study
*B. pseudomallei* 1026b Δ*bpss0711*Δ*bpsl2179*+pAlr-comp	Unmarked deletions of *bpss0711 and bpsl2179* carrying complementation replicating vector pAlr-comp; no d-alanine requirement	This study
*B. pseudomallei* 1026b Δ*bpss0711*Δ*bpsl2179*Δ*flgK*	Unmarked deletions of *bpss0711*, *bpsl2179*, and *flgK*, d-alanine requiring and non-motile	This study
*B. pseudomallei* 1026b Δ*bpss0711*Δ*bpsl2179*Δ*flgK*+pAlr-comp*flgK*	Unmarked deletions of *bpss0711*, *bpsl2179*, and *flgK* carrying complementation replicating vector pAlr-comp *flgK*; no d-alanine requirement and motile	This study
*B. mallei* ATCC 23344 Δ*bma1575*	Unmarked deletion of *bma1575*, d-alanine requiring	This study
*E. coli* MB1910	*endA* derivative of *E. coli* TG1 with a *dadX* allele insertionally inactivated by a kanamycin resistance (Km^R^) determinant flanked by *frt* sites	Michael Benedik
*E. coli* MB2786	Contains an *alr* allele insertionally inactivated by a tetracycline resistance (Tc^R^) determinant flanked by *frt* sites	Michael Benedik
*E. coli* ALA1	Alanine racemase deficient *E. coli* strain MB1910	This study
*B. mallei* ATCC 23344 Δ*bma1575*+pAlr-comp	Unmarked deletion of *bma1575* carrying complementation replicating vector pAlr-comp; no d-alanine requirement	This study

**Table 2:** Plasmids used in this study.

Vector	Relevant features and use	Reference
pET17*alr*	pET17 containing *E. coli alr*	Michael Benedik
pCP20	Encodes *flp* recombinase	[56]
pCR2.1-TOPO®-*bpss0711*	pCR2.1-TOPO® containing *B. pseudomallei bpss0711*	This study
pCR2.1-TOPO®-*bpss0711-*F2	pCR2.1-TOPO® containing *B. pseudomallei bpss0711*-F2 that has a 250 bp longer upstream sequence than *bpss0711*	
pCR2.1-TOPO®-*bpsl2179*	pCR2.1-TOPO® containing *B. pseudomallei bpsl2179*	This study
pCR2.1-TOPO®-*bma1575*	pCR2.1-TOPO® containing *B. mallei bma1575*	This study
pMo130	Suicide vector for allelic exchange in *Burkholderia*; pUC19 *ori*, RK2 *ori*T, *xylE*, *sacB*, Km^R^, used to construct pAlr-allex	[12]
pMo146	Source of Δ*flgK*	[12]
pMo168	Replicative vector for *Burkholderia*; *or*ipBBR1, *mob*+, *xylE*, Km^R^, used to construct pAlr	[12]
pMo173	Source of *flgK* for complementation	[12]
pAlr-allex	Suicide vector for allelic exchange in *Burkholderia* alr mutants; pUC19 *ori*, RK2 *ori*T, *xylE*, *sacB*, *bpsl2179*	This study
pAlr-comp	Replicative vector for *Burkholderia*; *ori*pBBR1, *mob*+, *xylE*, *bpsl2179*	This study

**Table 3:** Primers used in this study.

Primer name	Sequence
BPSS0711-down	attcgaggaggacgacatggaccga
BPSS0711-up	aacccatgcaacaaagaataggaca
BPSS0711orfdel-F	ctcgccccatcgtcgcccgcgcgctcgcccagccggtgcg
BPSS0711orfdel-R	cgcaccggctgggcgagcgcgcgggcgacgatggggcgag
BPSS0711-F	tgcgcaggtttcgtgcccgc
BPSS0711-R	gcgcgtcggacgcggctc
BPSS0711-F2	tgatcggatggtccgggcca
BPSL2179 down	accgcgagcagcatcgcgagccggttctgt
BPSL2179 up	accgcctgcaggatcgcgccttcggatagc
BPSL2179-down-HindIII	accgcgagcagcatcgcgagaagcttctgt
BPSL2179F2-2	atgatgaggcagccgacgaccttgatcgaa
BPSL2179orfdel-F	cgatttccgccacgatccacgtcgcgccgcgcgtgcccgt
BPSL2179-up-SmaI	accgcctgcaggatcccgggttcggatagc
BPSL2179orfdel-R	acgggcacgcgcggcgcgacgtggatcgtggcggaaatcg
BPSL2179R2-2	cgatctgcgcgagcgactgcagcagcagcg
Δflgk-US-NheI	gtcgtcagtagctagcctcgtcacccgcattctcgatgtcgacg
ΔflgK-DS-HindIII	gtacgatcgacaagcttcggtgcccgcctgcggcgcgggcgtcaccgtg

### Analysis of nucleotide and encoded amino acid sequences of putative alanine racemase genes of *B. pseudomallei* and *B. mallei*

We searched the annotated genomes of *B. pseudomallei* K96243 and *B. mallei* ATCC 23344 for previously identified putative alanine racemase genes. To search for potentially unannotated alanine racemase genes, we used the nucleotide sequences of the *alr* and *dadX* genes from *E. coli* strain DH10B [37] in the tBLASTx program on the NCBI website (http://www.ncbi.nlm.hin.gov) to probe the sequenced genomes of *B. pseudomallei* K96243 and *B. mallei* ATCC 23344. An analysis of amino acid sequence homology between the putative alanine racemase proteins encoded by *B. pseudomallei bpsl2179* and *bpss0711*, *B. mallei bma1575*, and several other known alanine racemases was performed using http://www.ch.embnet.org/software/ClustalW.html.

### Construction of the alanine racemase deficient mutant strain *E. coli ALA1*

The genome of *E. coli* has two known alanine racemase genes, *alr* and *dadX*. We obtained the following constructs from Michael Benedik: a clone of the *alr* gene designated pET17*alr*; *E. coli* MB1910, an *endA* derivative of *E. coli* TG1 with a *dadX* allele that is insertionally inactivated by a kanamycin resistance (Km^R^) determinant flanked by *frt* sites; and *E. coli* MB2786, which contains an *alr* allele insertionally inactivated by a tetracycline resistance (Tc^R^) determinant flanked by *frt* sites. To construct *E. coli* ALA1, we first transferred the Tc^R^-marked *alr* allele from MB2786 into MB1910 by phage P1 transduction and demonstrated that the resulting transductant was both Km^R^ and Tc^R^ and also able to form colonies on LB agar supplemented with DL-alanine but not on un-supplemented LB agar, consistent with inactivation of both *alr* and *dadX*. Next, we removed the Km^R^ and Tc^R^ determinants by introducing an Ap^R^ temperature-sensitive plasmid pCP20 [56] that encodes *flp* recombinase, which catalyzes recombination between the *frt* sites flanking the Km^R^ and Tc^R^ determinants. To complete the construction of strain ALA1, we eliminated pCP20 by overnight growth at a non-permissive temperature (37°C), leaving the *alr* and *dadX* alleles with single inactivating *frt* sites but without the Tc^R^ or Km^R^ determinants.

### Complementation of *E. coli* ALA1 with cloned alanine racemase genes from *B. pseudomallei*

We designed the primer pairs BPSL2179F2/BPSL2179R2 and BPSS0711F/BPSS0711R to PCR amplify *bpsl2179* and *bpss0711*, respectively, along with their putative promoters. Since the region of the *B. mallei* chromosome that contains *bma1575* is nearly identical to the region containing *bpsl2179* in *B. pseudomallei*, the primer pair BPSL2179F2/BPSL2179R2 was also used to amplify *bma1575* with its putative promoter. Additionally, primer pair BPSS0711-F2/BPSS0711-R was used to amplify a fragment that contained a 250 bp longer upstream sequence than that amplified by primer pair BPSS0711-F/BPSS0711-R. Touchdown PCR was performed using Ex Taq DNA polymerase as previously described [12]. PCR amplicons were cloned into pCR2.1-TOPO® using conditions specified by the manufacturer (Invitrogen, Carlsbad, CA). The *bpsl2179*, *bpss0711*, *bpss0711*-F2, and *bma1575* genes from these clones were sequenced at the University of Colorado Cancer Center DNA Sequencing and Analysis Core Facility and compared with the annotated genome sequence of *B. pseudomallei* strain K96243 and *B. mallei* ATCC 23344, respectively. To assess the function of the putative alanine racemase proteins encoded by *bpsl2179*, *bpss0711*, and *bma1575*, we transformed the pCR2.1-TOPO®-*bpsl2179*, pCR2.1-TOPO®-*bpss0711*, pCR2.1-TOPO®-*bpss0711*-F2, and pCR2.1-TOPO®-*bma1575* clones into *E. coli* ALA1 made competent by chemical treatment, as described previously [57]. *E. coli* ALA1 transformants containing pCR2.1-TOPO® -*bpsl2179*, pCR2.1-TOPO®-*bpss0711*, pCR2.1-TOPO®-*bpss0711*-F2, or pCR2.1-TOPO®-*bma1575* were selected on LB agar without added d-alanine. Representative colonies were picked and retested for their ability to grow on LB agar with and without d-alanine at 1 mM.

### Construction of alanine racemase deficient mutants of *B. pseudomallei* and *B. mallei*

To generate unmarked, in-frame deletions in the *bpsl2179* and *bpss0711* chromosomal genes of *B. pseudomallei*, we first used PCR to construct clones with the desired Δ*bpsl2179* and Δ*bpss0711* alleles flanked by approximately 1000 bp segments of the contiguous upstream and downstream flanking regions from the *B. pseudomallei* K96243 chromosome. We cloned each of these constructs separately into the mobilizable suicide vector pMo130, and we used the resulting clones with previously described allelic exchange protocols to substitute the Δ*bpsl2179* and Δ*bpss0711* alleles for their corresponding wild type alleles in the chromosomes of *B. pseudomallei* strains K96243 and 1026b [12]. In addition, we used the pMo130 clone carrying Δ*bpsl2179* with similar methods to substitute the Δ*bpsl2179* allele for the wild type *bma1575* allele in *B. mallei* strain ATCC 23344. This was possible because the chromosomal region in *B. mallei* that contains *bma1575* has almost complete nucleotide sequence identity with the corresponding chromosomal segment from *B. pseudomallei* K96243. Additional experimental details are provided in the following paragraphs.

To construct an in-frame deletion of *bpsl2179*, we used the primer pair BPSL2179-up and BPSL2179-orfdelR with chromosomal DNA from *B. pseudomallei* K96243 to PCR amplify a fragment containing approximately 1000 bp from the upstream flanking region of *bpsl2179* linked to the first 30 bp from the 5′ end and the last 30 bp from the 3′ end of the *bpsl2179* coding region. We used primers BPSL2179-down and BPSL2179-orfdelF (which is complementary to BPSL2179-orfdelR) in a similar manner to PCR amplify a second fragment containing the first 30 bp from the 5′ end and the last 30 bp from the 3′ end of the *bpsl2179* coding region linked to approximately 1000 bp from the downstream flanking region of *bpsl2179*. The fragments produced by these first two PCR reactions were then used as templates in a second PCR with primers BPSL2179-up and BPSL2179-down to generate Δ*bpsl2179*, an amplicon containing the 60 nucleotide long, internally in-frame deleted variant of the *bpsl2179* coding sequence flanked by approximately 1000 bp long wild type upsteam and downstream sequences. The resulting Δ*bpsl2179* amplicon was cloned into pCR2.1-TOPO®, generating pCR2.1-TOPO®-Δ*bpsl2179*. pCR2.1-TOPO®-Δ*bpsl2179* was digested with *Hind*III and *Xba*I to release the Δ*bpsl2179* fragment, which was Klenow-treated as described by the manufacturer (Invitrogen, Carlsbad, CA) and cloned into *Sma*I digested pMo130 (15). The resulting plasmid pMo130-Δ*bpsl2179* was introduced into *B. pseudomallei* strains K96243 and 1026b by biparental mating as previously described [12]. Co-integrants were selected on LB agar containing 100 µg/ml kanamycin, 10 mM d-alanine, and 50 µg/ml zeocin (to counterselect the *E. coli* donor strain). Co-integrants that expressed the *xylE* reporter gene from pMo130 were recognized as yellow colonies after being sprayed with a mist of pyrocatechol, as previously described [12]. For resolution of the co-integrants, individual colonies were inoculated into YT broth, grown for a minimum of 4 hours, diluted, and plated on YT agar containing 15% sucrose, as previously described [12], plus 10 mM d-alanine. A similar procedure was also performed with *B. mallei*; however, for selection of co-integrants, 50 µg/ml polymyxin B was used instead of zeocin to counterselect the *E. coli* donor.

An in-frame deletion of *bpss0711* was constructed in the same manner as Δ*bpsl2179*; however, primer pair BPSS0711-up and BPSS0711-orfdelR and primer pair BPSS0711-down and BPSS0711-orfdelF were used in PCR with chromosomal DNA to amplify the upstream and downstream fragments linked to the internally deleted *bpss0711* allele, respectively. A second PCR, using primers BPSS0711-up and BPSS0711-down and the fragments produced in the first PCR as templates produced Δ*bpss0711*, which was cloned into pCR2.1-TOPO®. Following digestion by *Hind*III and *Xba*I, the Δ*bpss0711* fragment was Klenow-treated as described above and subcloned into *Sma*I digested pMo130 (15). The resulting pMo130-Δ*bpss0711* was then introduced into the Δ*bpsl2179* single mutants of *B. pseudomallei* K96243 and 1026b, and also into wild type *B. mallei* ATCC 23344, by biparental matings, and co-integrants were selected and resolved as described above. Resolved mutants were tested for growth on LB agar with and without 10 mM d-alanine to identify the putative Δ*bpsl2179/*Δ*bpss0711* double mutants of *B. pseudomallei* K96243 and 1026b and the putative Δ*bma1575* single mutant of *B. mallei* ATCC 23344, all of which were shown to require the exogenous d-alanine for growth. Presence of the Δ*bpsl2179* and Δ*bpss0711* alleles in the *B. pseudomallei* double mutant, presence of the Δ*bpsl2179* allele in the *B. mallei* single mutant, and absence of the corresponding wild type alleles was confirmed by PCR tests using either primer sets BPSL2179-up and BPSL2179-down or BPSS0711-up and BPSS0711 down, as appropriate.

### Complemention tests with alanine racemase deficient mutants of *B. pseudomallei* and *B. mallei*

To perform complementation tests, we first constructed a derivative of the mobilizable, replication competent plasmid pMo168 [12] with a copy of *bpsl2179* from *B. pseudomallei* K96243. Briefly, pMo168 was digested with *Spe*I and *Xba*I to remove the *aphA* cassette that confers resistance to kanamycin, yielding the digested pMo168Δ*aphA* fragment. Plasmid pCR2.1-TOPO® -*bpsl2179* was digested with *Spe*I and *Xba*I, and the fragment containing *bpsl2179* was ligated with the digested pMo168Δ*aphA* fragment, yielding pALR-comp. The resulting plasmid was transformed into *E. coli* ALA1 made competent by chemical treatment and plated on LB agar to confirm that the cloned *bpsl2179* directed production of active alanine racemase. Following incubation at 37°C, the resulting colonies were tested to confirm the presence of pALR-comp, and the *bpsl2179* allele from pALR-comp was sequenced to confirm its identity with the wild type *bpsl2179* allele. Plasmid pALR-comp was then transferred from *E. coli* ALA1(pAlr-comp) into the Δ*bpsl2179/*Δ*bpss0711* double mutants of *B. pseudomallei* K96243 and 1026b and the Δ*bma1575* single mutant of *B. mallei* ATCC 23344 by triparental matings with DH5α(pRK2013) [58]. Putative transformants were selected on LB agar containing 50 µg/ml zeocin for matings with *B. pseudomallei* recipients or 50 µg/ml polymyxin B for matings with *B. mallei* recipients, and the presence of pALA1 in transformant colonies was confirmed by spraying them with pyrocatechol to detect the *xylE* reporter and sub-culturing them onto LB agar and LB agar supplemented with 50 µg/ml zeocin or 50 µg/ml polymyxin B to confirm the intrinsic antibiotic resistance phenotypes of the *B. pseudomallei* and *B. mallei* parental strains, respectively.

### Quantitative determination of d-alanine requirement for growth of alanine racemase deficient mutants of *B. pseudomallei* and *B. mallei* on solid medium

Wild type and isogenic alanine racemase deficient mutant strains of *B. pseudomallei* and *B. mallei* were grown in LB broth supplemented with 10 mM d-alanine overnight at 37°C. Overnight cultures were subcultured into LB broth supplemented with 10 mM d-alanine at an initial OD_600_ = 0.05 and grown to log phase (OD_600 nm_ = 0.2). Bacteria from log phase cultures were collected by centrifugation, washed with LB broth, resuspended in LB broth to the original volume, and inoculated on LB agar supplemented with 0 mM, 1.25 mM, 2.5 mM, 5 mM, or 10 mM d-alanine. Plates were incubated at 37°C for 36–48 hours and growth was observed.

### Effects of d-alanine on growth and viability of alanine racemase deficient mutants of *B. pseudomallei* and *B. mallei* in liquid medium

Wild type and isogenic alanine racemase deficient mutant strains were grown overnight at 37°C in LB containing 10 mM d-alanine. Cultures were centrifuged, supernatants removed, and the pellets were washed with LB broth and inoculated at an initial OD_600 nm_ = 0.1 into LB broth with or without 10 mM d-alanine. Cultures were incubated at 37°C with shaking, and samples were removed every 30 minutes for the first 4 hours, then hourly up to 7 hours and again at 24 hours for measurement of OD_600 nm_ and determination of viable counts on LB agar containing 10 mM d-alanine. To assess the consequences of shorter periods of d-alanine deprivation, each the alanine racemase deficient mutant strains of *B. pseudomallei* and *B. mallei* was inoculated into replicate cultures in LB medium without d-alanine as described above, and at 30 minute intervals from time zero up to 120 minutes d-alanine at 10 mM was added back to single cultures. During further incubation, samples were removed from all of the replicate cultures at 30 minute intervals up to 180 minutes and then at hourly intervals up to 7 hours for measurements of growth (OD_600 nm_) and of viability on LB agar with 10 mM d-alanine.

### Isolation of murine peritoneal macrophages

C57BL/6 mice were bred and murine peritoneal macrophages were prepared under protocols 56409(05)1B and 56410(05)1E, which were approved on 5/18/10 and 5/5/10, respectively, by the University of Colorado Denver Animal Care and Use Committee. Peritoneal macrophages were harvested from mice 4 days after intraperitoneal inoculation of 1 mg/ml sodium periodate as described [59]. The peritoneal exudate cells were re-suspended in RPMI 1640 medium (Sigma-Aldrich, St. Louis, MO) supplemented with 10% heat-inactivated fetal bovine serum (BioWhittaker, Walkersville, MD), 15 mM Hepes, 2 mM L-glutamine, 1 mM sodium pyruvate (Sigma-Aldrich), and penicillin/streptomycin at 100 U/ml and 100 µg/ml, respectively (RPMI^+^; Cellgro, Manassas, VA). This medium was supplemented with d-alanine at 5 mM where specified. The peritoneal exudate cells were seeded in flat-bottom 96-well plates at a density of 3_×_10^5^ cells per well for macrophage killing assays. The macrophages were selected by adherence after 24 hours of culture at 37°C in a 5% CO_2_ incubator. Just prior to infection, the macrophages were washed in pre-warmed RPMI containing 5 mM D-alanine.

### Macrophage killing assays

Macrophage killing capacity was assessed by a gentamicin or a kanamycin protection assay using a modification of a protocol described for *B. mallei* [60]. *B. mallei* strains were grown overnight in LB and sub-cultured to OD_600 nm_ = 0.6 in LB. *B. pseudomallei* strains were grown overnight in LB to an OD_600 nm_ = 11. The bacteria were collected by centrifugation and opsonized by suspending them for 20 minutes at 37°C in RPMI^+^ medium containing 10% normal mouse serum and 5 mM d-alanine. The opsonized wild type or isogenic alanine racemase deficient strains of *B. mallei* and *B. pseudomallei* were added at a multiplicity of infection of 200 to macrophage monolayers in RPMI^+^ containing d-alanine at 5 mM, and the infected monolayers were incubated for 2 hours with the *B. mallei* strains or for 3 hours with the *B. pseudomallei* strains to permit phagocytosis of the opsonized bacteria by the macrophages [60], [61]. Extracellular bacteria were removed by washing the monolayers with pre-warmed RPMI^+^ medium containing 6 µg/ml of gentamicin for experiments with *B.mallei* or 250 µg/ml of kanamycin for experiments with *B. pseudomallei*. After removal of the extracellular bacteria, the average multiplicity of infection was determined to be 10. Fresh RPMI^+^ medium, with or without d-alanine at concentrations indicated in the text, and with 6 µg/ml of gentamicin for experiments with *B.mallei* or 250 µg/ml of kanamycin for experiments with *B. pseudomallei* was added to the monolayers. After incubation for 0 hours, 3 hours, 4 hours, or 5 hours for experiments with *B. pseudomallei* and 0 hours or 4 hours for experiments with *B. mallei*, five replicate cultures of the infected macrophages were lysed with 1% Triton X-100 in phosphate buffered saline (PBS), and the numbers of viable intracellular bacteria were determined by plating on LB agar for the wild type stains or on LB agar supplemented with 5 mM d-alanine for the alanine racemase deficient strains. The percent survival was calculated as (cfu at t_n_/cfu at t_0_) ×100.

### Electron Microscopy

Following phagocytosis of wild type or isogenic alanine racemase deficient strains of *B. pseudomallei* and *B. mallei*, the macrophages were plated as previously described [59] at a density of 4×10^5^ per chamber of a 8-well Permanox Labtek chamber slide system (Nalgene Nunc International, Rochester, NY). The cells were fixed in 2.5% glutaraldehyde in phosphate buffer, pH 7.4. The specimens were post-fixed in 1% osmium tetraoxide, treated with uranyl acetate, dehydrated in ascending ethanol series, and infiltrated with Embed 812. Ultrathin sections were examined for the presence and morphological integrity of intracellular bacteria in a FEI Technai 62 electron microscope operated at 80 kV.

### Construction and complementation of a *B. pseudomallei* flagellar mutant using alanine racemase as the selectable genetic marker

To demonstrate the utility of alanine racemase as a selection marker for genetic manipulations in *Burkholderia* spp., we constructed the allelic exchange vector pAlr-allex by replacing *aphA* of pMo130 [12] with *bpsl2179* from *B. pseudomallei* K96243. Briefly, pMo130 [12] was digested with *Spe*I and *Xba*I to remove the *aphA* cassette, yielding the pMo130Δ*aphA* fragment. To clone the wild type *bpsl2179* allele into pMo130Δ*aphA*, pCR2.1-TOPO® -*bpsl2179* was digested with *Spe*I and *Xba*I and the resulting fragment containing *bpsl2179* was ligated with the restriction-digested pMo130Δ*aphA* fragment, yielding pALR-allex. The pALR-allex plasmid was transformed into chemically competent alanine racemase deficient *E. coli* strain ALA1 and plated on LB agar without added d-alanine. Growth of the transformants at 37°C demonstrated expression of the *bpsl2179* gene in pALR-allex. The presence of pALR-allex in the transformants was confirmed, and the region flanking *bpsl2179* was sequenced to confirm the predicted nucleotide sequence of the wild type *bpsl2179* allele.

We wished to demonstrate directly that pALR-allex can be used for allelic exchange protocols in pathogenic *Burkholderia* spp. Toward that end, we first subcloned the *ΔflgK* allele from pMo146 [12] into pALR-allex. Briefly, we PCR amplified *ΔflgK* using primers Δflgk-US-NheI and ΔflgK-DS-HindIII, digested the resulting amplicon with *Nhe*I and *Hind*III, and ligated it with into linearized pALR-allex generated by treating pALR-allex with *Nhe*I and *Hind*III. The resulting plasmid, pALR-allexΔ*flgK*, was transformed into *E. coli* strain ALA1 made competent by chemical treatment, and transformants were selected by growth on LB agar without added d-alanine. Next, pALR-allexΔ*flgK* was introduced into the alanine racemase deficient Δ*bpss0711*Δ*bpsl2179* double mutants of *B. pseudomallei* K96243 and *B. pseudomallei* 1026b by triparental matings, as described above. Co-integrants were selected by growth on LB agar without added d-alanine, and resolved co-integrants were subsequently selected by growth on LB agar containing 15% sucrose plus 10 mM d-alanine. We determined the *ΔflgK* or *flgK*+ genotype of resolved co-integrants by screening individual colonies for their non-motile or motile phenotypes, respectively.

For *in-trans* complementation of Δ*flgK B. pseudomallei* mutants, we subcloned the wild type *flgK* gene from pMo173 [12] into the pALR-comp plasmid described previously. The *flgK* gene with its predicted promoter were excised from pMo173 [12] by digestion with *Nhe*I and *Hind*III and the resulting fragment was ligated into *Nhe*I- and *Hind*III-digested pALR-comp to generate pALR-comp-*flgK*. pALR-comp-*flgK* was introduced by triparental matings into the Δ*flgK* mutants of *B. pseudomallei* K96243 and 1026b strains described above, and the resulting transconjugant colonies were selected by growth on LB agar with added d-alanine and screened for restoration of the motile phenotype.
